# Lead Poisoning among Male Juveniles Due to Illegal Mining: A Case Series from South Africa

**DOI:** 10.3390/ijerph18136838

**Published:** 2021-06-25

**Authors:** Thokozani Patrick Mbonane, Angela Mathee, André Swart, Nisha Naicker

**Affiliations:** 1 Department of Environmental Health, Faculty of Health Sciences, University of Johannesburg, Johannesburg 2000, South Africa; Angie.Mathee@mrc.ac.za (A.M.); andres@uj.ac.za (A.S.); nnaicker@uj.ac.za (N.N.); 2 Environment and Health Research Unit, South African Medical Research Council, Johannesburg 2000, South Africa; 3 School of Public Health, Faculty of Health Sciences, University of Witwatersrand, Johannesburg 2000, South Africa

**Keywords:** illegal mining, elevated blood levels, artisanal and small-scale gold mining, para-occupational, environmental health

## Abstract

Illegal mining is a major public health and societal concern. Recent scientific evidence indicates elevated blood–lead levels in illegal gold miners and associated communities. Yet, there is little research in this regard from low- to middle-income countries (LMICs), where illegal mining is growing. This case series is extracted from a cross-sectional study of lead exposure in incarcerated juveniles in greater Johannesburg. From survey records (blood–lead levels and questionnaires), three males had elevated blood–lead levels and presented with health conditions and behavioural problems putatively linked with lead poisoning. Based on the record review, all three juveniles were in a secure facility due to illegal mining-related activities. All three cases had high blood–lead levels and demonstrated a tendency toward aggressive or violent behaviour. They also presented with conditions associated with lead poisoning, such as anaemia, respiratory illness, abdominal disorders, and musculoskeletal conditions. Juveniles involved in illegal mining are at risk of exposure to heavy metals such as lead, and there is a need for relevant preventative action and health care programmes in this group.

## 1. Introduction

Environmental and para-occupational lead exposure is an under-recognized societal issue in low- to middle-income countries [1]. Children are particularly affected, including adolescent males [2]. Blood–lead levels (BLLs) have been linked to societal and behavioural issues such as aggression, anti-social behaviour, delinquency, and violent or criminal behaviour [2,3,4,5,6,7]. Research has highlighted elevated BLLs in adolescent males who are prone to involvement in acts of violence or criminal [4,5]. Elevated BLLs have also been linked to the number of arrests and the type of crime committed [8]. Studies from high-income countries have shown that children in juvenile systems have elevated blood–lead levels compared to children who have never been in conflict with the law [5].

Involvement in informal or artisanal mining has been associated with lead exposure [9,10]. Artisanal gold mining is regarded as an illegal economic activity in many low- to middle-income countries, including South Africa [11,12]. Elevated blood–lead levels have been found in as many as 92.5% of children aged under six years within an artisanal mining community [10]. Boys residing in communities near artisanal and small-scale gold mining activities are at greater risk of having high blood–lead levels than girls [13]. Similar findings were found near the lead–zinc mine, where eight children had BLLs exceeded 427.8 μgdL^−1^ [14]. However, reductions in BLLs have been seen in a community where safer artisanal mining practices have been adopted [15].

Artisanal mining has been growing in several African countries, with growing concerns over the health of those involved [16]. South Africa has a long history of gold mining, increasing artisanal gold mining in recent decades [17]. Despite this, there is a paucity of information on exposure to toxic substances and the health of this vulnerable group that often includes adolescents and children. In South Africa, children and young adults found guilty of criminal acts are confined to secure facilities [18]. They may receive access to services such as education, recreation, and limited public-health programmes (curative health only) as part of their rehabilitation programmes before being released to a parent or guardian [19]. These services are designed to address mental, social, and community factors that contribute to criminal behaviour, but seldom address preventable environmental exposures as contributors to violent or criminal behaviour.

This study presents a series of three cases of elevated blood–lead levels related to artisanal mining in male juveniles held in secure facilities in South Africa.

## 2. Materials and Methods

From October 2020 to February 2021, venous blood samples were collected by a qualified and registered nurse from three children/juveniles at the correctional centres. Approximately 50–100 μL of blood were collected into an anticoagulant ethylene diamine tetra–acetic acid sterile test tube and transported on the same day to the laboratory. The blood samples were divided in the laboratory to determine blood–lead levels and haemoglobin. Information on the criminal behaviour of participants was retrieved from their records at the incarceration facilities. A semi-structured questionnaire was administered to participants to obtain information on socio-demographic factors, self-reported health issues, and environmental exposures. The Youth Self-Report scale was used to screen for tendencies to aggression or violent behaviour [20,21].

The study was approved by the Faculty of Health Sciences, Research Ethics Committee from the University of Johannesburg. The ethics approval number is (REC-241112-035I). The researchers sought consent from young males of 18 years or older. The parents and legal guardians of the young males were also approached for consent. The three cases reported here are from an ongoing study [22].

## 3. Findings

### 3.1. Case Report 1

An 18-year-old male had a blood–lead level of 48.11 µg/dL, with a haemoglobin level of 7.9 g/dL (severe anaemia). Before coming to the incarceration centre, he had lived in an informal settlement with a communal water supply. He was of Mozambican birth and raised by a single parent (his mother). He started working as an artisanal gold miner at the age of 15 years in South Africa. He was a digger (colloquially referred to as “moles”), who went underground to dig for gold and handled explosives for underground blasting. From time to time, he served as a “drainer” as well. A drainer is responsible for refining gold from the dirt retrieved from underground. He was arrested and incarcerated for illegal gold mining in Randfontein.

The participant indicated that he was required to work for periods of up to two weeks underground without coming to the surface. While underground, he was not provided with masks or any other personal protective gear to provide protection against dust or contaminated air. He usually used a cloth or discarded personal respiratory safety equipment that he had found. While underground, he ate only canned foods and bread. Occasionally, he received food from a “runner” (also called messengers) responsible for removing the waste material generated underground. He smoked marijuana and used other illicit substances.

There were no health outcomes recorded in his file; however, he complained of severe headaches, abdominal pain, constipation, painful muscles, weak joints, and a persistent cough. He has shown tendencies of violent behaviour in the last six months. He indicated that he had been shoved or pushed, hit, emotionally/verbally abused, and robbed, mainly underground. Lastly, he had regularly threatened someone with a sharp object to protect himself and the gold that he had discovered while underground.

### 3.2. Case Report 2

An 18-year-old male with blood–lead levels of 45.87 µg/dL and Hb of 7.1 g/dL (severe anaemia). Prior to his incarceration, he had lived in an informal settlement with a communal water supply. He had been born in Mozambique and lived with his unemployed mother until relocating to South Africa. He was recruited by an older male family friend to smuggle explosives for use in the informal gold-mining sector. In time he became a messenger and a drainer.

He had never been given personal protective gear. He used discarded safety goggles, boots and cloth as a mask; most of these materials he had found the refinery sites where discovered gold was smelted prior to sale.

There was no medical history captured in the participant’s file. He indicated having experienced chest pains, constipation, persistent cough, joint weakness, and muscle pains in the last 12 months and feeling tired most of the time since he had been in the facility. He admitted to being violent towards other gangs in the area where they mined gold. In the last six months, he has verbally abused, threatened to hurt, robbed, and used a sharp object to assault someone from a rival group.

### 3.3. Case Report 3

A 15-year-old immigrant boy had a blood–lead level of 35.76 µg/dL and haemoglobin of 8.6 g/dL, classified as severe anaemia. He had been orphaned at a young age and left his home in Mozambique due to physical abuse from a relative. Prior to being incarcerated, he had lived in a South African informal settlement with his brothers, who had introduced him to illegal gold mining. He had been working as an explosives handler and as a digger when needed.

The most prolonged period during which he remained underground was one week. He was not provided with personal protective clothing and ate only canned foods and bread while underground.

He did not report any medical conditions at the time of entry into the detention centre, but during the recent interview complained of itchy skin, pain in his fingers, and watery eyes after working with chemicals. He did not report any significant violent behavioural tendencies, apart from quickly becoming irritated. He reported having emotionally abused others during the six months before the interview.

## 4. Discussion

This case series gives insight into various environmental, social, and health risks faced by illegal adolescent gold miners in South Africa. With blood–lead levels of 35.76, 45.87, and 48.11 µg/dL, which exceed the current reference level of the Centers for Disease Control in the USA—of 5 µg/dL—by factors between 7 and 10, all three cases had been highly exposed to environmental lead. Their work in the field of artisanal mining is most likely the source of their lead exposure. The three participants involved informal gold-mining processes such as digging, transporting food supplies into the underground mining areas, and processing the soil retrieved from underground to extract gold residues (as a drainer). The drainer is the most exposed to heavy metals and other chemicals used in the gold-extraction process. This process involves the use of sodium cyanide, sulphuric acid, and nitric acid. All three cases reported a lack of personal protective equipment when underground or involved in other mining processes.

Previous studies have related high blood–lead levels to illegal mining or other artisanal activities [10,23,24], including a fatal lead-poisoning case [25]. According to CDC guidelines, the three individuals should be on a treatment programme for lead poisoning. The cases variably reported a range of non-specific ill-health symptoms that have been associated with lead exposure, including pain and weakness in their muscles and joints, constipation, severe headache, and abdominal pain.

Lead poisoning is a known neurotoxicant that can influence behaviour in particular ways, due to its ability to impair the nervous system [26]. All three cases showed signs and tendencies toward aggression or violent conduct, which have been linked to elevated lead levels [2,4,27]. Two participants have threatened to hurt, robbed, and assaulted someone with a sharp weapon, while all three have verbally/abused someone recently. This behaviour was similar to evidence from a non-exposed population with elevated blood–lead levels in South Africa [4]. Nkomo and colleagues found that young males with elevated blood–lead levels were prone to violent behaviour such as assault, robbery, and assault using a weapon with the intent to cause harm [4].

The working conditions of the cases were also highly insanitary, including a lack of water, sanitation, ventilation, and waste disposal systems. Such conditions have the potential to be highly detrimental to the subjects’ health [28]. In addition, the accounts given to the interviewer illustrate the marginalization and vulnerability of the cases, for example, in terms of being in a foreign country (they were all of Mozambican origin and had come to South Africa as adolescents or children), their young age and work as child labourers in the artisanal mining sector, and their subjection to threats and violence underground over the gold they had found.

Young artisanal miners are a highly marginalized and vulnerable group regarding their abysmal and perilous working conditions and their hazardous environmental exposures, social dangers, and compromised health. They find themselves beyond the safety net of public health and social systems. There is a greater need for collaboration between different sectors such as health, social development, educational, mineral resources, economic development, and criminal justice. The action might include introducing safer mining practices at a small-scale level through regulating artisanal mining, developing a lead-poisoning screening tool and implementation of cases to the public health officials. Meanwhile, the juveniles who are in the secure facilities should be educated on the dangers of illegal mining. Incarcerated juveniles receive numerous services, such as counselling, the opportunity to continue with their studies (from primary to secondary levels), and to take skills training programmes. Once the juveniles have left the facilities they may be aided go back to school and access the social grant system. In addition, health education on lead hazards amongst public officials dealing with male juveniles and pro-environmental intervention is required to address environmental-related activities linked to societal and health issues in LMICs.

## 5. Conclusions

The study has highlighted the impact of illegal gold mining on young males’ livelihoods, especially their education, health, and violent behaviour. The young males had blood–lead levels that require public health action, either curative or preventive. The juveniles were involved in illegal activities for survival that contribute to high blood–lead levels. There is a need for a holistic approach to address societal issues such as crime. This report identifies the need for lead poisoning monitoring and a surveillance programme on the exposed population or communities.

## Data Availability

The data presented in this study can be made available on request from the corresponding author.
